# Compliant Motion Planning Integrating Human Skill for Robotic Arm Collecting Tomato Bunch Based on Improved DDPG

**DOI:** 10.3390/plants14050634

**Published:** 2025-02-20

**Authors:** Yifan Zhang, Yajun Li, Qingchun Feng, Jiahui Sun, Chuanlang Peng, Liangzheng Gao, Liping Chen

**Affiliations:** 1 School of Agricultural Engineering, Jiangsu University, Zhenjiang 212013, China; vishkcc@163.com; 2 Intelligent Equipment Research Center, Beijing Academy of Agriculture and Forestry Sciences, Beijing 100097, China; liyj@nercita.org.cn (Y.L.); sjiahui9@126.com (J.S.); pcl109969@163.com (C.P.); glzzz2024@163.com (L.G.); 3 Beijing Key Laboratory of Intelligent Equipment Technology for Agriculture, Beijing 100097, China

**Keywords:** deep reinforcement learning, demonstration learning, path planning, tomato bunch collection, robotics

## Abstract

Dexterous manipulation and gradual placement are crucial for preserving fruit integrity during harvesting. Addressing the limitations of conventional path planning methods in learning manual compliant skills, we propose a novel method for tomato bunch collection that integrates human-robot skill transfer with Deep Deterministic Policy Gradient (DDPG). In our method, a demonstrator manually guided the robotic arm using an existing tomato collection mechanism, with spatial trajectories recorded as demonstration paths. We then developed an enhanced DDPG-Z model that incorporates human skill replay for pre-training, expert reward regression loss to stabilize pre-training, and dynamic step-length returns to balance short- and long-term rewards. Subsequently, the agent was trained to minimize the deviations of key points between the demonstration paths and actual paths, effectively approximating human operations. In a highly realistic simulation environment, our method achieved a 25% improvement in convergence speed, a 10.3% increase in post-convergence reward, and a 51.3% boost in destination accuracy compared to the case without the demonstrations, whereas classical models such as DDPG, SAC (Soft Actor-Critic), and TD3 (Twin Delayed Deep Deterministic Policy Gradient) failed to converge within the prescribed episodes. This work provides valuable insights for enhancing the compliant operational performance of agricultural robots.

## 1. Introduction

Tomatoes are widely cultivated fruits around the world, characterized by their delicious taste and nutritional benefits. However, fresh tomato picking, sorting, and packing are typically labor-intensive tasks. Especially in recent years, with the increasing yield per unit area (about 40 kg/m^2^) [1], in China, the labor costs for the selective harvesting of tomatoes in greenhouses have reached 40% of the total production costs [2]. Given that intelligent harvesting robots have unique advantages in autonomously engaging in complex operations, they can mechanize selective harvesting tasks while saving labor costs, so they have become a global hotspot for agricultural robotics research [3,4].

During the collection of fresh fruits, minimizing damage to the tender pericarp and fruit abscission from the cluster is a prerequisite for ensuring their edible quality. However, current research on fresh fruit harvesting robots mainly focuses on picking [5,6], ignoring the importance of collection operations in maintaining the quality of fruit bunches. In the conventional strategy [7,8], the robotic arm, after completing the picking, directly transfers fruit bunches to the collection area. The fruit bunches enter the basket after a short free fall, which risks damaging the delicate fruits. Thus, the above method has significant shortcomings in terms of the non-destructive collection of ripe fruits and the orderly arrangement of fruit bunches. In traditional manual harvesting, compliant and dexterous collection techniques as a prior knowledge developed through long-term training guide the operator in gently and gradually placing bunches into the basket following a sequence from the bottom of the fruit to the stalk. This collection method, which takes into account the vulnerability of fresh fruits, provides an important reference for guiding the robotic arm to perform human-like compliant operations. Therefore, in order to enable the robotic arm to draw on the prior knowledge accumulated in the traditional manual collection and to reproduce compliant operations, there is an urgent need to develop a path planning method with learning capability. This is of key importance for improving the overall performance of tomato-harvesting robots.

Among the currently available path planning methods, traditional methods, such as the artificial potential field algorithm [9], A-star algorithm [10,11], and Rapidly-exploring Random Tree (RRT) [12], have been widely used to generate collision-free paths. However, they suffer from significant deficiencies in replicating complex human operations. This phenomenon arises primarily from two factors. First, these traditional methods are designed to generate feasible paths and struggle to directly integrate the implicit features of human skills, such as flexibility and compliance, into their optimization objectives. Second, they lack the capability to autonomously learn from empirical data, which makes it difficult to leverage existing prior knowledge effectively. These limitations hinder their application in learning human-like skills, thus driving the development of data-driven and learning-based path planning methods, with imitation learning (IL) and reinforcement learning (RL) being the most representative approaches. IL methods can learn the corresponding control strategies and motion characteristics from expert demonstration data [13,14]. However, due to the high reliance on expert data, the performance of agents trained by IL is usually limited to the expert level only and has poor generalization to situations beyond expert data. In practice, demonstration samples rarely cover the entire state space and often contain suboptimal examples [15].

In contrast, the “trial-and-error” mechanism of RL allows an agent to learn by interacting with the environment and continuously adjusting its strategy to maximize rewards based on feedback, which demonstrates a generalization performance superior to IL. Furthermore, with the rise of deep reinforcement learning (DRL) [16,17] in recent years, its powerful feature extraction and decision-making capabilities have led to significant progress in path planning for robotic arms in high-dimensional continuous spaces. Lin et al. [18] proposed a robotic arm path planning method combining recurrent neural networks and DDPG for guava harvesting robots, capable of quickly planning collision-free paths with a success rate of 90.9%. Evan et al. [19] extended the problem to multi-armed robots by combining SAC and HER (Hindsight Experience Replay) for path planning, and the improved algorithm was able to plan the shortest path for any start and endpoints. These DRL-based robotic arm path planning studies [18,19,20,21,22] have undoubtedly laid a solid foundation for realizing the compliant collection of fruit bunches. Furthermore, DRL algorithms, especially DQN (Deep Q-Network) [16], DDPG [23], SAC [24], etc., can effectively incorporate demonstrations and improve model performance due to their off-policy nature, which allows these algorithms to learn from experiences generated by other policies. Hester et al. [25] proposed DQfD (Deep Q-learning from demonstrations), a DRL algorithm that introduces a small amount of artificial prior data, which significantly improves the learning of Atari games and alleviates the problem of scarcity of samples at the beginning of RL. However, DQfD is limited to discrete action spaces and does not apply to robotics. Therefore, Vecerik et al. [26] proposed DDPGfD (DDPG from demonstrations), which outperformed DDPG by shaping rewards in a series of sparse reward insertion tasks by the robotic arm. Subsequently, another study [27] introduced an optimized DDPGfD for more complex multistep, multi-objective tasks. The model achieved a 99% success rate on the 3-block stacking task with fully sparse rewards by introducing HER, a behavioral cloning loss filter, and other techniques. Liu et al. [28] used DDPG combined with demonstrations for the path planning task of the harvesting robotic arm and verified the effect of the ratio of expert data to interaction samples on the model performance. The model reached a 72.1% success rate when this ratio was dynamically reduced from 0.45 to 0.35. In summary, it is feasible to utilize a deep reinforcement learning approach that incorporates demonstrations for tomato bunch collection path planning. Notably, these studies typically employed sparse reward mechanisms that only required the robotic arm to reach the target position without collision but without specifying the concrete shape of the motion path. However, the collection operation requires not only precise positioning but also dexterousness and compliance similar to manual operation, introducing new constraints and challenges on the path shape for motion planning. Therefore, a strategy that combines demonstrations with a well-designed reward function is necessary to enable the robotic arm to accurately reproduce the manual collection behaviors.

In conclusion, this study introduces a compliant motion planning approach for the harvesting robotic arm engaged in the fruit bunch collection, which is based on demonstrations and deep reinforcement learning. The objective is to achieve compliance and orderliness in the collection process of tomato bunches, avoiding damage to the fruits due to impact forces from rigid motion and sudden release. The main contributions are summarized as follows:

- Establishing a human–robot skill transfer mechanism using demonstration data. Initially, a set of demonstration paths was acquired by manually dragging the robotic arm to place tomato bunches. These paths were subsequently processed and converted into a format suitable for reinforcement learning. Thereafter, reward functions reflecting the characteristics of compliant collection operation were designed in combination with the shape of the demonstration paths to instruct the robotic arm to learn human skills;
- The DDPG algorithm has been enhanced through the integration of several key components: demonstration data, expert reward regression (ERR) loss, dynamic step-length return (DSR), and prioritized experience replay (PER) mechanism. This amalgamation culminates in the proposal of the DDPG-Z model. The objective of this refined model is to more effectively absorb manual compliant and dexterous operation strategies, thereby enhancing the efficiency and stability during the robotic arm’s execution of fruit bunch collection tasks.

## 2. Materials and Methods

### 2.1. Tomato Bunch Collection Mechanism

**Figure 1:**
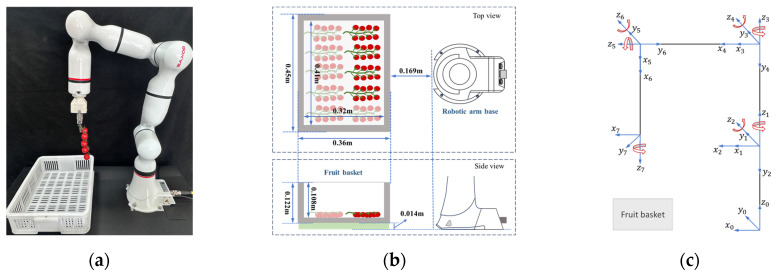
Overview of tomato collection mechanism: (**a**) tomato collection mechanism; (**b**) the relative position between the robotic arm base and the fruit basket; (**c**) kinematic model of the robotic arm based on improved Denavit–Hartenberg (D–H) method.

Figure 1 shows our tomato collection mechanism. The mechanism consists of two main components: a fruit basket and a robotic arm. This study employs a seven-degree-of-freedom robotic arm, which can effectively mimic the dexterity of the human arm and is thus suitable for more complex tasks. The robotic arm consists of seven links and seven independently actuated joints, with the end-effector connected to and considered as part of the seventh link. We constructed a kinematic model of the robotic arm using the improved Denavit–Hartenberg (D–H) method (Figure 1c), and the D–H parameters of the robotic arm are presented in Table 1. The outer dimensions of the fruit basket are 0.45 × 0.36 × 0.122 m, and the inner dimensions are 0.42 × 0.32 × 0.108 m. We determine the relative position of the fruit basket based on the operating area of the robotic arm and preset a total of 10 placement positions in 2 rows and 5 columns according to the pre-measured dimensions of the basket and tomato bunches. Furthermore, to avoid unnecessary collisions, we adjust the position and posture of the robotic arm as illustrated in Figure 1a, and then use this configuration as the initial point for the collection task.

### 2.2. Compliant Path Planning of Tomato Bunch Collection

#### 2.2.1. Demonstration Paths Acquisition and Processing

(1) Demonstration paths acquisition

According to whether or not additional equipment is used, the acquisition methods of demonstration data in imitation learning can be categorized into indirect demonstration and direct demonstration. Indirect teaching requires remapping the data acquired through visual observation [29] or wearable devices [30] to the robot’s actions, which is a complicated process and may have conversion errors. Direct teaching, on the other hand, can directly obtain demonstration data matching its structure from the robot’s sensors, and the acquisition process is relatively simple and fast, with typical methods such as kinesthetic teaching [31] and teleoperation teaching [32]. The xMateER3Pro robotic arm used in this study is equipped with the drag function. By obtaining information about the motion of the robotic arm under kinesthetic control, it was used as demonstration data to guide learning.

**Figure 2:**
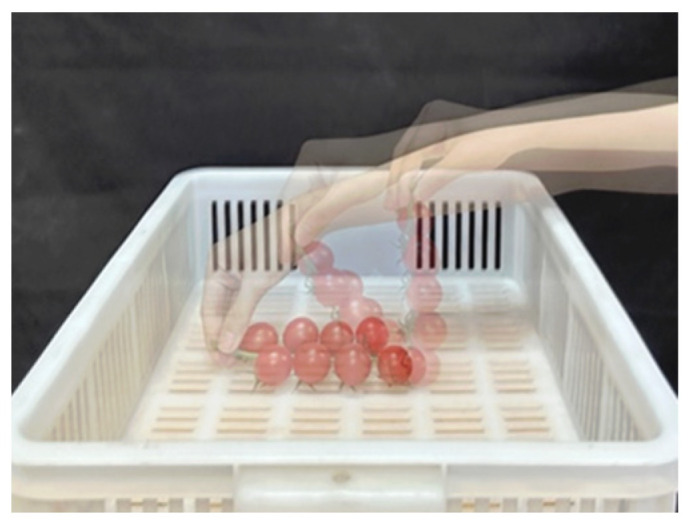
Manual placement operation.

Manual collection of fruit bunches can be divided into two main processes: transferring and placing. First, transfer the tomato bunch to the top of the placement area, then gradually lower the entire bunch in the order from the bottom of the fruit to the stalk (Figure 2), which is the process that we need the robotic arm to learn and reproduce.

In the position and posture shown in Figure 1a, a demonstrator dragged the robotic arm to replicate the operation of manually placing fruit bunches, and the end-effector position data was recorded in real time. To facilitate the later screening of data, the frequency of acquisition was 20 Hz, and the acquisition was repeated three times for each placement position.

During the process of kinesthetic teaching, manual operations often introduce random fluctuations and instability, resulting in noisy trajectories. To mitigate these issues and enhance the quality of demonstration data, data smoothing and adjustment are crucial steps. Initially, our data preprocessing involved two key transformations: First, we converted the robot arm’s base coordinate system to align with the world coordinates of the simulation environment. Second, we changed the units from millimeters to meters to ensure consistency with the simulation framework. To further refine the demonstration paths, we employed cubic spline interpolation. This method was chosen for several reasons: It is a simple yet widely adopted technique that requires relatively low computational overhead. More importantly, cubic spline interpolation effectively smooths the paths by generating continuous curves with smooth transitions. By flexibly adjusting the path based on selected data points, it preserves the overall shape and trend of the original trajectory while eliminating high-frequency noise caused by hand jitter. As a result, the processed paths are smoother with more uniform data points, providing higher-quality data for model training.

Given that the utilization of cubic spline interpolation for path adjustment is not the primary focus of this study, we provide only a brief overview of its underlying principles. Set the coordinates of points on the path to be $\left(x_{i},y_{i},z_{i}\right)$, cubic Spline will calculate the interpolation of three-dimensional coordinates respectively, the following is introduced as an example of the horizontal coordinate $x_{i}$. Given a set of time series $\left(t_{0},t_{1},\ldots ,t_{n}\right)$ and horizontal coordinates $\left(x_{0},x_{1},\ldots ,x_{n}\right)$, the goal of cubic spline interpolation is to find a cubic polynomial $S_{i}\left(t\right)$ in each interval $\left[t_{i},t_{i+1}\right]$ (i=0,1,…,n−1):

(1) $$S_{i}\left(t\right)=a_{i}{\left(t-t_{i}\right)}^{3}+b_{i}{\left(t-t_{i}\right)}^{2}+c_{i}\left(t-t_{i}\right)+d_{i},$$

where $S_{i}\left(t_{i}\right)=x_{i}$, $S_{i}\left(t_{i+1}\right)=x_{i+1}$, and $a_{i},b_{i},c_{i},d_{i}$ are coefficients to be determined, which can be solved according to the conditions in [33].

**Figure 3:**
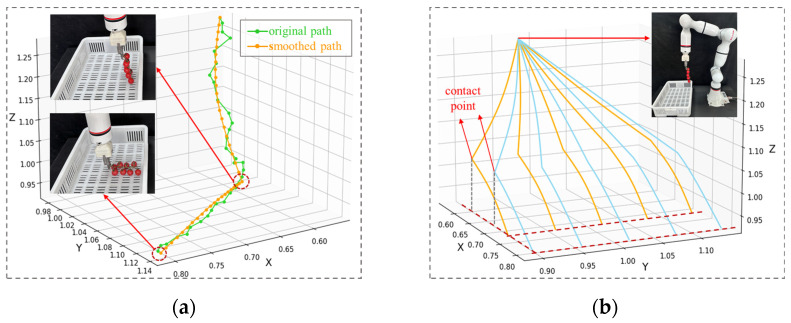
Demonstration paths: (**a**) comparison of the path obtained by dragging the robotic arm with the path generated by the cubic spline; (**b**) ten demonstration paths generated by the cubic spline.

Figure 3a shows the comparison of one of the paths before and after adjustment. Although the path captured by the manual kinesthetic control of the robotic arm (green curve) is not smooth enough, it exhibits a distinct characteristic that resembles the shape of a deformed “L”. The inflection point on the curve corresponds to the moment when the bottom of the fruit bunch makes contact with the fruit basket (hereinafter referred to as the contact point), and the destination of the curve indicates that the fruit bunch has been completely placed into the basket. Smoothed paths (orange and blue curves) reinforce this characteristic and make the position of all contact points and destinations more accurate. Figure 3b shows ten demonstration paths generated from the cubic spline.

(2) Conversion of demonstration paths

For the agent to understand the demonstration data, the paths need to be transformed into reinforcement learning transition tuples $\langle s_{i},a_{i},r_{i},s_{i+1},done\rangle$, all of which are stored in the experience replay buffer for the agent to sample and learn. The specific definitions of the agent’s state, action, and environmental rewards will be elaborated in Section 2.2.2. Here, it is sufficient to clarify that the action of the agent represents the change in position of the end-effector. The method of converting a demonstration path into reinforcement learning transition tuples can be summarized as follows: first, a trajectory tracking method is used to compute the action required for the robotic arm to move from the current position to the next position on the demonstration path; then, the computed action is input into the reinforcement learning environment of the robotic arm and the corresponding reward as well as the next state information is obtained from the environment, thus a transition tuple $\langle s_{i},a_{i},r_{i},s_{i+1},done\rangle$ is obtained; finally, the above steps are repeated until the robotic arm reaches the end point. This method is more accurate and convenient than manual computation and also verifies the validity of the demonstration data in the simulation environment simultaneously.

Action computation is a pivotal component of the entire data transformation process, with its accuracy significantly influencing the subsequent model training. As previously defined, the action represents the incremental displacement of the end-effector. Consequently, the most straightforward method for acquiring the action sequence is using the difference between neighboring positions on the demonstration path. However, in practical operation, slight deviations often exist between the actual and theoretical positions of the robotic arm’s end-effector. For instance, when designating the starting point A of the demonstration path as the initial position of the end-effector, the actual position of the end-effector may be A’. Although this error is seemingly negligible, it will accumulate and magnify over the course of action execution, ultimately leading to significant deviations between the robotic arm’s motion path and the demonstration path. To address this issue, we employed a trajectory tracking method. Specifically, the action $a_{i}$ required for the robotic arm to move from its current position to the next position on the demonstration path is computed by subtracting the current actual position $p_{i}$ of the end-effector from the next target position $path_{i+1}$. By dynamically incorporating the actual position of the end-effector, this approach effectively mitigates the accumulation of errors, thereby enabling the robotic arm to more accurately follow the demonstration path. The detailed process is described in Algorithm 1.
**Algorithm 1:** conversion of demonstration paths**Input:** ten demonstration paths
**Output:** ten demo files
1:      **for** n←1 to 10 **do**
2:              Read the n-th demonstration path into the array path
3:              Calculate the length l of the array path
4:              Reset the environment, obtain the initial state $s_{0}$, and extract $p_{0}$ from $s_{0}$
5:              $a_{0}=path_{1}-p_{0}$
6:              Initializes an empty list demo
7:              **for**
 i←0 to l−2 **do**
8:                       perform action $a_{i}$, and obtain the reward $r_{i}$, while the state turns to $s_{i+1}$
9:                       Append the transition $\langle s_{i},a_{i},r_{i},s_{i+1},done\rangle$ to the list demo
10:                      **if** i<l−2 **then**
11:                             Extract $p_{i+1}$ from $s_{i+1}$
12:                             $a_{i+1}=path_{i+2}-p_{i+1}$
13:                      **else**
14:                             Set done=True
15:                      **end if**
16:                      **if** done is True **then**
17:                             Save the contents of the list demo to a demo file
18:                      **end if**
19:                      $s_{i}\leftarrow s_{i+1}$
20:              **end for**
21:              Close the environment
22:       **end for**

#### 2.2.2. Reinforcement Learning Interaction Environment

(1) State and action space

For the fruit bunch collection task, our goal is to train an agent that can learn dexterous manipulation and gradual placement demonstrated by human skills, to minimize fruit damage from falls and impacts, thereby improving the overall operational performance of the robotic arm. Therefore, the state observed by the agent can be described as:

- Position of the end-effector *p* = [*x*, *y*, *z*];
- Radians of rotation for the seven joints of the robotic arm rad *q* = [α_1_,…, α_7_].

To summarize, at time step *t*, the state can be comprehensively described as: $s_{t}=\left[p,q\right]\in S$.

The action of the agent includes position increments and posture changes of the end-effector. Since the end-effector needs to maintain a vertical downward orientation during the fruit bunch collection process, we set it to a fixed posture. Therefore, the actual action is represented as $a_{t}=\left[\Delta x,\Delta y,\Delta z\right]$. To avoid drastic positional changes, the action increments are constrained to be within the range of [−0.02, 0.02] m.

(2) Reward functions

In this research, we aim to guide the robotic arm to reinforcement learning through a human–robot skill transfer mechanism. Consequently, the shaping of the reward function is closely related to the demonstration data. This is primarily reflected in two aspects: firstly, the reward function should be capable of reflecting and generalizing the common characteristics of the demonstration data to clarify that the learning goal of the agent is the human skill of gently placing fruit bunches; secondly, the reward function should not merely fit the demonstration data nor simplistically compare with expert behaviors, to avoid transforming the learning process into imitation learning, which could lead to difficulties in generalizing the model when faced with new situations. Therefore, the design of the reward function needs to strike a balance between these two key points, providing a clear learning goal for the robotic arm.

Based on the path characteristics shown in Figure 3, we divided the robotic arm movement process into two stages: end-effector approaching the contact point and approaching the destination, and designed reward functions for each stage to guide the robotic arm to learn the human operation of collecting fruit bunches. In the first stage, the reward function $r_{1}$ was a linear function of the Euclidean distance between the current position of the end-effector $p_{t}$ and the contact point $p_{c}$ (which can be calculated from the current destination):

(2) $$r_{1}=\lambda_{1}||p_{t}-p_{c}|{|}_{2}.$$

In the second stage, we employed the reward function $r_{2}$ to facilitate the end effector’s approach to the destination while avoiding collisions of the robotic arm with the fruit basket:

(3) $$r_{2a}=\left\{\begin{array}{l} \lambda_{2}||p_{t}-p_{goal}|{|}_{2}\cdot I\left(condition_{1}\right)\\ \lambda_{3}||p_{t}-p_{goal}|{|}_{2}\cdot I\left(\neg condition_{1}\right) \end{array}\right.,$$

(4) $$r_{2b}=\lambda_{4}\cdot I\left(condition_{2}\right)\;,$$

(5) $$r_{2}=r_{2a}+r_{2b}.$$

Here, $\lambda_{1,2,3,4}$ are weighting factors requiring precise adjustment. $p_{goal}$ represents the destination. I is an indicator function that equals 1 when the condition is met and 0 otherwise. $I\left(condition_{1}\right)$ is satisfied when the Euclidean distance between the end-effector and the destination is within the desired threshold. For certain hazardous situations, we imposed an additional penalty $r_{2b}$ on the agent; the $I\left(condition_{2}\right)$ is met when the end-effector collides with the fruit basket.

(3) Simulation environment

**Figure 4:**
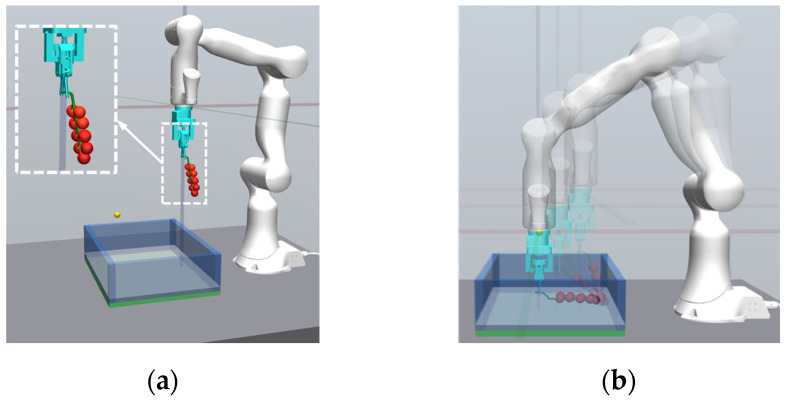
Simulation environment based on the real tomato harvesting robot: (**a**) overview of the simulation environment; (**b**) the placement process of tomato bunch in the simulation environment.

To accurately simulate the interaction between the robotic arm and the physical environment during the fruit bunch collection process, we constructed a simulation environment (Figure 4) based on the physics engine simulator MuJoco (Multi-Joint dynamics with Contact). Additionally, we utilized MuJoco’s “mocap” (motion capture) functionality to control the position and posture of the robotic arm’s end-effector. As shown in Figure 4a, the mocap object is fixed to the end-effector, and its position is determined by the intersection of three coordinate axes.

In the simulation environment, the robotic arm, the fruit bunch, and the fruit basket form the core components. To enhance the realism of the simulation environment and the accuracy of the simulation, the following settings were implemented:

- The relative position of the robotic arm to the fruit basket was consistent with the actual tomato collection mechanism shown in Section 2.1, and key parameters of the robotic arm (such as dimensions, weight inertia, friction, and joint velocities) were extracted from the Robot Universal Description Format (URDF) file for simulation;
- The destination of the end-effector was marked by a yellow dot;
- The tomato bunch consisted of two parts: the fruit stalk and the fruit itself, with size parameters set based on statistical data;
- The fruit stalk was fixed to the end-effector of the robotic arm and was immobile; the fruit part could rotate around the stalk by a certain angle to simulate the flexibility of a real tomato bunch.

#### 2.2.3. Optimized DDPG for Tomato Bunch Collection Task

To learn human dexterous collection skills and reduce the ineffective exploration of the agent during the early stages of training, we have improved upon the DDPGfD method based on the research [26] and proposed a decision-making model for the compliant path planning of fruit bunch collection. In terms of network structure, our proposed model DDPG-Z inherits the four neural networks of DDPG. The actor network $\pi_{\theta }\left(s\right)$ and the critic network $Q_{\mu }\left(s,a\right)$ output the action and the value of the state-action pair, respectively, and they each possess a target network, $\pi_{\theta \prime }\left(s\right)$ and $Q_{\mu \prime }\left(s,a\right)$, respectively.

Pre-training is a mainstream practice in demonstration-based reinforcement learning, commonly employing a two-step paradigm that includes offline learning and online learning [34]. In the offline learning phase, the agent is prohibited from interacting with the environment and instead directly utilizes demonstration data for pre-training. In the online learning phase, the agent is treated as a conventional reinforcement learning agent, interacting with the environment to generate data, which is then added to the experience replay buffer for sampling and learning. The conventional DDPG algorithm consists solely of the online learning phase, where the agent begins learning from a randomized policy and typically requires a substantial amount of interaction data for networks to update and converge. To enhance learning efficiency, we introduced a pre-training procedure to the conventional DDPG, guiding the agent to learn human collection skills through demonstration data.

In the offline learning phase, the actor network and critic network were updated solely based on demonstration data, without updating two target networks, aiming to stabilize training and reduce computational complexity. Additionally, we designed a novel expert reward regression (ERR) loss function tailored for demonstration data, which facilitated the rapid and adequate learning of effective information about manual operation strategies. In the online learning phase, we focused on updating the networks using data from the agent’s interaction with the environment, discarding the strategy of sampling a mixture of demonstration and interaction data that was employed in previous research [26,27]. This approach aimed to encourage the agent to move beyond the confines of the demonstration policies, exploring potentially superior behaviors, thereby reducing the risk of overfitting and aligning the model’s training more closely with real-world application scenarios. Furthermore, we introduced a dynamic step-length return (DSR) mechanism to balance short-term and long-term returns while improving sample efficiency. Lastly, we specified that two target networks were updated once per episode during the online training phase.

In summary, the model was pre-trained and trained online by the aforementioned approach. Throughout the learning process, the model maintained two distinct replay buffers, one for storing demonstration data and the other for interaction data. Additionally, prioritized experience replay (PER) is applied to all data.

(1) DDPG with PER

DDPG [23] combines the advantages of DPG [35] (Deterministic Policy Gradient) and DQN [16,36] methods, constructing a framework that enables efficient learning in continuous action spaces. To further enhance data efficiency, PER was introduced into DDPG, assigning priorities to all experiences based on their relevance to the learning objectives, thereby accelerating the algorithm’s absorption of critical knowledge [37].

The priority $p_{i}$ of the sample *i* is associated with the TD error and the Q value of taking the optimal action output in the current state [26], aiming to give precedence to experiences that could lead to significant learning updates:

(6) $$p_{i}={\delta_{i}}^{2}+Q_{\mu }{\left(s_{i},\pi_{\theta }\left(s_{i}\right)\right)}^{2}+\epsilon_{min},$$

where $\epsilon_{min}$ is a very small constant to ensure that the sampling probability is not zero for all experiences. $\delta_{i}$ represents the TD error of the sample *i*, i.e., the difference between the actual Q value $Q_{\mu }\left(s_{i},a_{i}\right)$ and the target Q value $G_{i}$.

The target Q value $G_{i}$ is calculated based on the two target networks:

(7) $$G_{i}=r_{i}+\gamma Q_{\mu^{\prime }}\left(s_{i+1},\pi_{\theta \prime }\left(s_{i+1}\right)\right),$$

where γ is a discount factor in the range 0 to 1, but usually close to 1.

The sampling probability $F\left(i\right)$ of the sample *i* in the experience replay buffer is positively correlated with its priority $p_{i}$:

(8) $$F\left(i\right)=\frac{p_{i}^{\alpha }}{\sum_{j}p_{j}^{\alpha }},$$

where *α* controls the influence of experience priority on the sampling probability; when *α* is 0, it degenerates into uniform random sampling.

For DDPG combined with PER, the critic network still employs the Mean Squared Error (MSE) function to minimize the TD error; however, to address the bias introduced by prioritized sampling, importance sampling weights $W_{i}$ are typically used to adjust the updates during the learning process:

(9) $$L_{critic-1}=\frac{1}{N}\sum_{i=1}^{N}W_{i}\cdot {\left(G_{i}-Q_{\mu }\left(s_{i},a_{i}\right)\right)}^{2},$$

(10) $$W_{i}={\left(\frac{1}{N\cdot F\left(i\right)}\right)}^{\beta },$$

where *β* influences the degree to which the weights correct for the bias.

The actor network is updated by the gradient ascent algorithm to find an action to maximize the expected return in a given state:

(11) $$\nabla_{\theta }J=\frac{1}{N}\overset{N}{\underset{i=1}{\sum }}\nabla_{\theta }\pi_{\theta }\left(s_{i}\right)\nabla_{a}Q_{\mu }\left(s_{i},\pi_{\theta }\left(s_{i}\right)\right).$$

In addition, the two target networks $\pi_{\theta^{\prime }}\left(s\right)$ and $Q_{\omega^{\prime }}\left(s,a\right)$ slowly track the parameters of two main networks using the soft-update method:

(12) $$\left\{\begin{array}{c} \theta^{\prime }\leftarrow \varphi \cdot \theta +\left(1-\varphi \right)\theta^{\prime }\\ \mu^{\prime }\leftarrow \varphi \cdot \mu +\left(1-\varphi \right)\mu^{\prime } \end{array}\right.\;,$$

where φ is a small update rate.

(2) Expert reward regression (ERR) loss

The goal of pre-training is to quickly and effectively absorb the policies contained within the demonstration data, which necessitates the development of a network update method suitable for this phase. In traditional DDPG, the value network’s iterative updates depend on the TD error computed by two target networks. However, updates to the two target networks tend to lag behind the online networks, which is more pronounced during the pre-training phase where the target networks remain stationary. Consequently, calculating the loss using under-trained target networks may result in low learning efficiency. To improve the learning effect of the initial policy and align it more closely with the human expert’s policy, we proposed an ERR loss for the critic network (Equation (13)). The ERR loss is designed to provide clearer learning signals during the pre-training phase and to avoid the instability introduced by the target network. Specifically, this loss is applied only to the demonstration data.

(13) $$L_{demo}=\frac{1}{N}\sum_{i=1}^{N}W_{i}\cdot {\left(r_{i}-Q_{\mu }\left(s_{i},a_{i}\right)\right)}^{2},$$

where $r_{i}$ is the true reward of the sample i.

(3) Dynamic step-length return (DSR)

The standard n-step return uses a fixed step size, which makes it difficult to balance short-term and long-term returns. To address this, we introduced a DSR strategy that increases the value of n dynamically during online training to enhance learning efficiency and model performance. In the early stages of online training, a smaller n value causes the agent to rely more on recent reward signals, reducing the estimation error of long-term returns and thus converging more quickly to locally effective policies. As the training progresses, the gradually increasing n value allows the agent to better estimate long-term returns. This helps the agent to escape from local optima, further optimize policies, and discover potential global optima behaviors. Furthermore, the 1-step return stabilizes training, so we use it as a complement to the DSR strategy, mixing these two return strategies during the online learning phase. Note that only 1-step return was used during the pre-training phase. Let $R_{n}$ be the current n-step return of the form:

(14) $$R_{n}=\sum_{k=0}^{n-1}\gamma^{k}r_{t+k}+\gamma^{n}Q_{\mu^{\prime }}\left(s_{t+n},\pi_{\theta^{\prime }}\left(s_{t+n}\right)\right),$$

*n* is the step length that changes dynamically with the number of training steps:

(15) $$n=\min\left(n_{max},n_{min}+cur_{step}|T_{n}\right),$$

where $n_{max}$ and $n_{min}$ denotes the maximum and minimum of *n*; $cur_{step}$ is the current training step, $T_{n}$ indicates the period of *n* value increase, | is the integer division symbol. The period, maximum, and minimum values need to be adjusted carefully and reasonably during training to achieve the best result.

The corresponding loss function is:

(16) $$L_{critic-n}=\frac{1}{2}\cdot W_{i}{\left(R_{n}-Q_{\mu }\left(s_{i},a_{i}\right)\right)}^{2}.$$

In summary, the loss function of the value network during the online learning phase is:

(17) $$L\left(\mu \right)=L_{critic-1}+L_{critic-n}.$$

**Figure 5:**
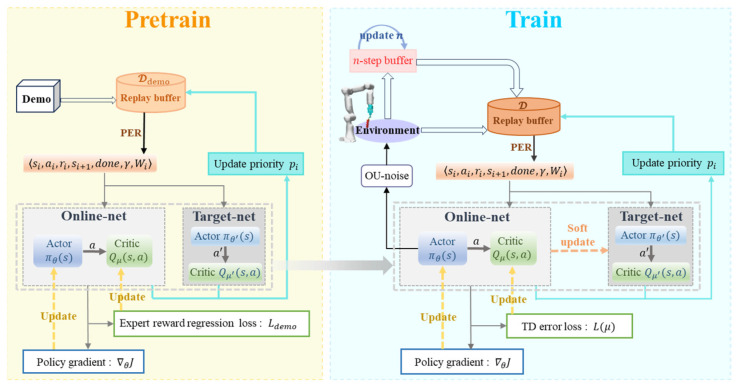
DDPG-Z framework. The model will be first pre-trained and then trained online. The model maintains two replay buffers, $D_{demo}$ and D, for storing demonstration data and interaction data, respectively. “PER” stands for Prioritized Experience Replay, and the priority $p_{i}$ is defined by Equation (6); the policy gradient $\nabla_{\theta }J$ is shown in Equation (11); Ldemo is defined by Equation (13); for $L\left(\mu \right)$, please refer to Equations (9), (16) and (17); and the description of the n-step can be found in Equations (14) and (15).

Figure 5 illustrates the framework of DDPG-Z model. Algorithm 2 shows the main training process of DDPG-Z.

**Algorithm 2:** major training process of DDPG-Z**Input:** ten demo files, the number of pre-training steps $n_{\mathrm{pre}}$, the number of training episodes $n_{\mathrm{train}}$ of training, and the number of network updates k for a single environment step.
**Output:** trained network models.
1:       Initialize four networks and parameters
2:       Initialize the Priority Experience Replay buffer $D_{demo}$ and D
3:       Initialize the DSR buffer and set the initial step-length n
4:       Load all demo data to $D_{demo}$
5:       **for** $i\leftarrow 1\;to\;n_{\mathrm{pre}}$ **do**
6:                Sampling a min-batch of N demo transitions $\langle s_{i},a_{i},r_{i},s_{i+1},done,\gamma ,W_{i}\rangle$
7:                Update the Critic Network: $L_{demo}$ ⇒ *ERR Loss*
8:                Update the Actor Network: $\nabla_{\theta }J$
9:                Update Sample Prioritization: $p_{i}$ ⇒*PER*
10:       **End for**
11:       **while** ($episode\leq n_{\mathrm{train}}$) **do**
12:                Reset the environment to obtain the initial observation $s_{0}$
13:                **while** (done is False) **do**
14:                         Select an action and add the noise
15:                         Execute the action $a_{t}$ to obtain the reward $r_{t}$ and the next state $s_{t+1}$
16:                         Store $\langle s_{t},a_{t},r_{t},s_{t+1},done,\gamma \rangle$ in D and DSR buffer
17:                         Process the data in DSR buffer and store them in D ⇒*DSR*
18:                         **for**
i←1 to k **do**
19:                                 Sampling a min-batch of N transitions $\langle s_{i},a_{i},r_{i},s_{i+1},done,\gamma ,W_{i}\rangle$
20:                                 Update the Critic Network: $L\left(\mu \right)$
21:                                 Update the Actor Network: $\nabla_{\theta }J$
22:                                 Update Sample Prioritization: $p_{i}$ ⇒*PER*
23:                         **End for**
24:                **End while**
25:                episode +=1
26:                Update step-length n ⇒*DSR*
27:                Update the parameters of two target networks
28:       **End while**

### 2.3. Tests

The test section is divided into two parts: ablation and comparative tests. All models were trained for 20,000 steps, which corresponds to 500 episodes.

#### 2.3.1. Ablation Experimental Setup

To evaluate the contribution of each module in the DDPG-Z model to the final performance, we conducted the following ablation tests to analyze the role of each optimization technique in our model.

- Standard model: the complete DDPG-Z model, including pre-training using demonstration data (pre-training steps set to 5000), applying expert reward regression (ERR) loss to the demonstration data, using the dynamic step-length return (DSR) strategy during online training and prioritized experience replay (PER) for all data;
- No Demo: the part of pre-training with demonstration data is removed, so there is also no need for ERR loss;
- No PER: all data is randomly sampled throughout the training process without updating the priorities of all samples;
- No DSR: the DSR mechanism is removed and only the 1-step return is used for all data throughout the training process;
- No ERR: update the critic network with regular TD error (Equation (9)) instead of ERR loss during the pre-training phase.

#### 2.3.2. Comparison Experimental Setup

We compared four DRL algorithms suitable for continuous action spaces, namely DDPG-Z, DDPG, SAC, and TD3 (Twin Delayed Deep Deterministic Policy Gradient). The latter three algorithms were rapidly implemented using the stable baselines3 learning library based on the PyTorch (v1.11.0) framework. To clarify the performance of each algorithm in the task of gradually placing tomato bunches, they were trained in the same environment using identical parameters.

#### 2.3.3. Performance Metrics

In evaluating the compliance and orderliness of the robotic arm placing tomato bunches, the distance between the end-effector and the contact point, as well as the distance to the destination, are two important criteria. Larger distance values typically indicate that the robotic arm deviates from the manual operation method during collection, while smaller distances suggest a collection effect closer to human-like performance. Additionally, the number of steps required for the end-effector to reach the destination reflects the efficiency of the robotic arm in placing the bunches. Therefore, in addition to the reward value, we introduced the following metrics to comprehensively evaluate the performance of the models:

- Minimum distance *d*_1_ between the robotic arm’s end-effector and the contact point, and its standard deviation *σ*_1_;
- Distance *d*_2_ between the end-effector and the destination at the end of a single episode, and its standard deviation *σ*_2_;
- Minimum number of steps $n_{step}$ required for the end-effector to reach and remain near the destination ($||p_{t}-p_{goal}|{|}_{2}\leq \delta_{goal}$), and its standard deviation $\sigma_{n}$, with $n_{step}$ set to 40 steps if the threshold condition is never met or if it is exceeded again after being reached;
- the success rate of the collection task, where the task is considered successful when both $d_{1}\leq \delta_{contact}$ and $d_{2}\leq \delta_{goal}$ are satisfied within a single episode, otherwise, it is deemed a failure. The values of $\delta_{goal}$ and $\delta_{contact}$ should be precisely set based on the size of the fruit basket and tomato bunches. Exceeding either threshold may result in interference between placement positions, increasing the risk of fruit bunch compression.

## 3. Results and Discussion

### 3.1. Ablation Tests

**Figure 6:**
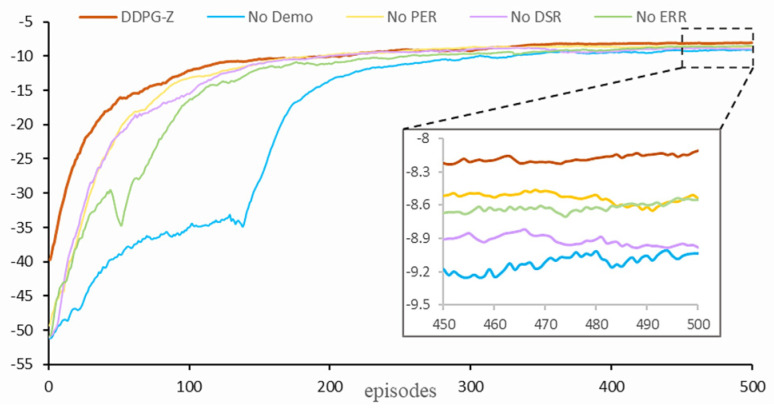
Average rewards for different algorithms and episode ranges.

Figure 6 displays the learning curves of five models—DDPG-Z, No Demo, No PER, No DSR, and No ERR—during the online training phase. In addition to the No Demo model (400 episodes), all other models reached convergence around 300 episodes, but the convergence performance of our model was overall superior to the other models. Firstly, in the early stage of online training, due to the limited quantity and poor quality of interaction data collected, the network parameters were not fully updated, and this phase was predominantly guided by the pre-training policy or initial policy. Therefore, we used the first 50 episodes to evaluate the effectiveness of the models’ pre-training policy or initial policy (Table 2). We found that the DDPG-Z model achieved the highest average reward among all models during the first 50 episodes, reaching −24.45, which was significantly higher than the other models. In contrast, the No Demo model, lacking a pre-training process, had the lowest reward value (−44.98) due to its initial random policy, and its curve exhibited significant oscillation before 200 episodes. The No EER model also experienced reward value oscillation in the early learning phase, indicating that the introduction of demonstration data and the ERR loss helped the model to learn a better policy during the pre-training phase, thereby achieving higher reward values in the early training stage. Secondly, in the later stage of training, i.e., the last 50 episodes, the reward values of the models reflected their final performance after convergence (Table 2). Similarly, our model achieved the highest reward value, with an average of approximately −8.18, which was a 10.3% improvement over the lowest (−9.12) No Demo model. Furthermore, the reward values of the other three models were intermediate, with differences of no more than 0.5. This result demonstrated that the introduction of demonstration data had the most significant impact on the model’s convergence performance.

To further compare the performance of each model, we recorded the results of 20 evaluation episodes in the same simulation environment (Table 3). The DDPG-Z model achieved the best scores across all four evaluation metrics. Specifically, the DDPG-Z model achieved a 100% collection success rate in the 20 evaluation episodes, which was at least 25% higher than that of the other models. In terms of the minimum distance to the contact point ($d_{1}$ = 0.027 ± 0.002 m), the distance to the destination at the end of the episode ($d_{2}$ = 0.019 ± 0.003 m), and the minimum number of steps required to stabilize within a certain range ($\delta_{goal}$) of the destination ($n_{step}$ = 25 ± 0 steps), DDPG-Z model obtained the smallest values with the lowest degree of data dispersion. This indicated that our model was able to complete the fruit bunch collection tasks stably and accurately. In contrast, the No Demo model had the lowest success rate of only 50%; its performance at the contact point ($d_{1}$ = 0.041 ± 0.006 m) and the destination ($d_{2}$ = 0.039 ± 0.011 m) was the worst. This suggested that No Demo model had a high risk of skewing and compression occurring to the fruit bunches during placement. Although the No Demo model was superior to the No DSR model in terms of $n_{step}$ (32.6 ± 6.7 steps versus 36.3 ± 3.2 steps), its overall performance was still the worst, further confirming that the introduction of demonstration data for pre-training had the most significant impact on improving model performance. We conducted a one-way analysis of variance (ANOVA) on the key metrics $d_{1}$, $d_{2}$, and $n_{step}$ for each of the five models over the 20 evaluation episodes to test whether the differences in performance among the models were statistically significant. The results showed that the F-values for all three metrics were significantly higher than the critical F-values and the *p*-values were well below the set level of significance (0.05). The results of this statistical analysis clearly demonstrate that the performance differences between the different models are statistically significant and not caused by random fluctuations.

**Figure 7:**
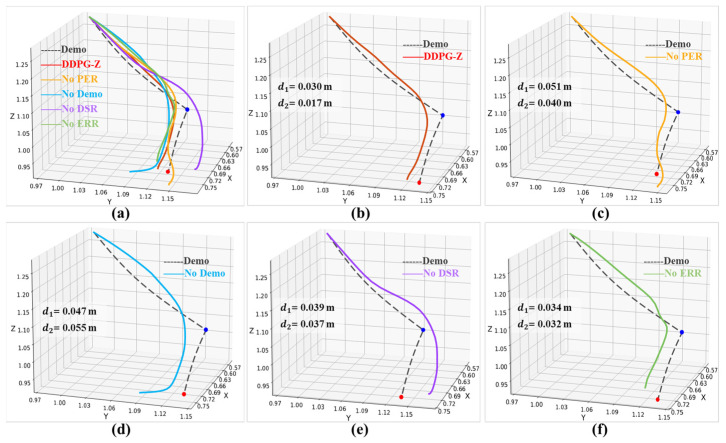
Comparison of the demonstration path and the paths planned by each model in ablation tests: (**a**) overview of the five model-planned paths and demonstration path; (**b**–**f**) individual comparisons of the DDPG-Z, No PER, No Demo, No DSR, and No ERR model-planned paths versus the demonstration path. The blue dot represents the contact point and the red dot represents the destination.

**Figure 8:**
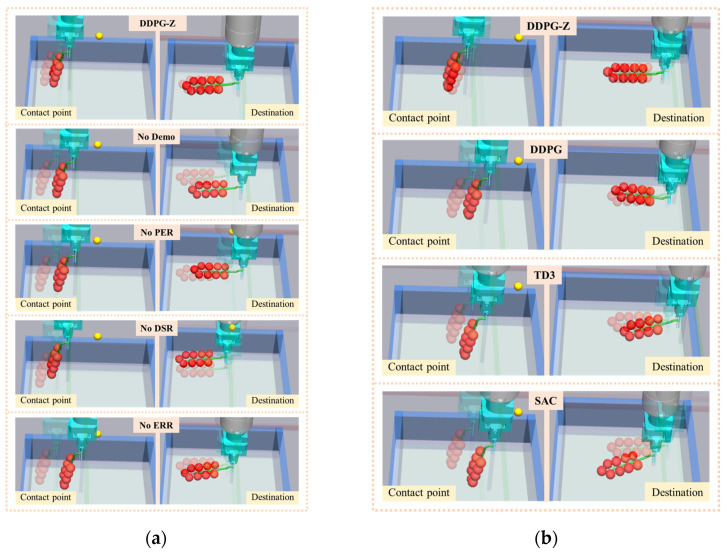
The placement effect of each model at the contact point and destination in the simulation environment: (**a**) the placement effect of each model in ablation tests; (**b**) the placement effect of each model in comparison tests.

Finally, to demonstrate the effect of different models in the compliant path planning for tomato bunch collection, we randomly selected a placement position and compared the paths planned by each model with the demonstration path. It is important to note that the demonstration path reflects manual operation and has high precision at the contact point and destination, with deviations from the desired values being extremely small (less than 0.001 m). Therefore, we used the demonstration path as a reference for comparison. The results are shown in Figure 7, where the black dashed line represents the demonstration path, and the blue and red dots indicate the desired contact point and destination, respectively. To avoid visual errors, we also calculated the deviations of each path from the contact point and destination ($d_{1}$ and $d_{2}$). The path planned by DDPG-Z most closely matched the shape of the demonstration path, with the smallest deviations from the contact point and destination. The path planned by No Demo showed a significant deviation near the destination, with the lowest similarity to the demonstration path. According to the definition of task success rate, the No Demo model failed to complete the collection task at this location. Figure 8a shows the effects of the robotic arm placing the fruit bunch along the aforementioned paths and the demonstration path in the simulation environment, depicting the situations at the contact point and destination, respectively. The lighter-colored fruit bunch in each figure represented the placement effect of the demonstration path. Whether at the contact point or the destination, the placement effect of the DDPG-Z model was the closest to that of the demonstration path. Other models, due to insufficient precision at the destination, were likely to experience collisions or encroach on other placement locations.

In summary, our model achieved the best results in terms of early-stage policy, convergence performance, stability, and simulation outcomes. The presence or absence of demonstration data had the most significant impact on the comprehensive performance of the model, thereby validating the importance of pre-training.

### 3.2. Comparison Tests

**Figure 9:**
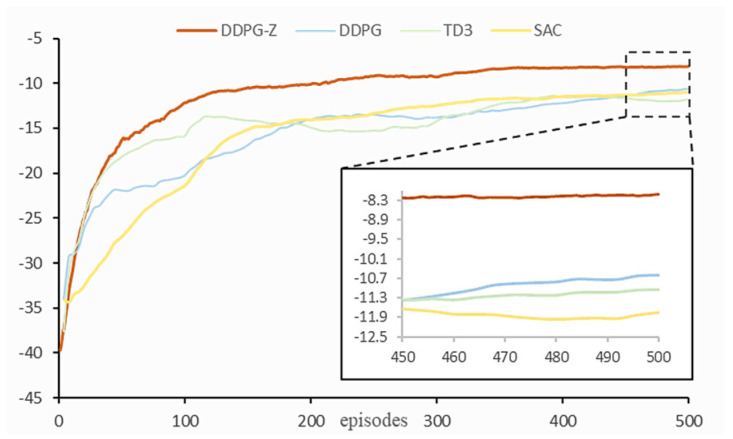
Learning curves for each model in comparison tests.

Figure 9 presents the learning curves of the DDPG, SAC, TD3, and DDPG-Z models. The results demonstrated that under the same shared parameters, our model significantly outperformed the other three models. Firstly, our model converged around 300 episodes, while the curves of the other three models only began to show signs of convergence towards the end of training, indicating a need for more training episodes to reach a steady state. Therefore, the convergence speed of our model was at least 40% faster. In the last 50 episodes, the average reward achieved by DDPG was −10.92, TD3 was −11.84, SAC was −11.20, and our model (−8.18) was significantly higher than these three models, with improvements of 25.1%, 30.9%, and 27%, respectively.

**Figure 10:**
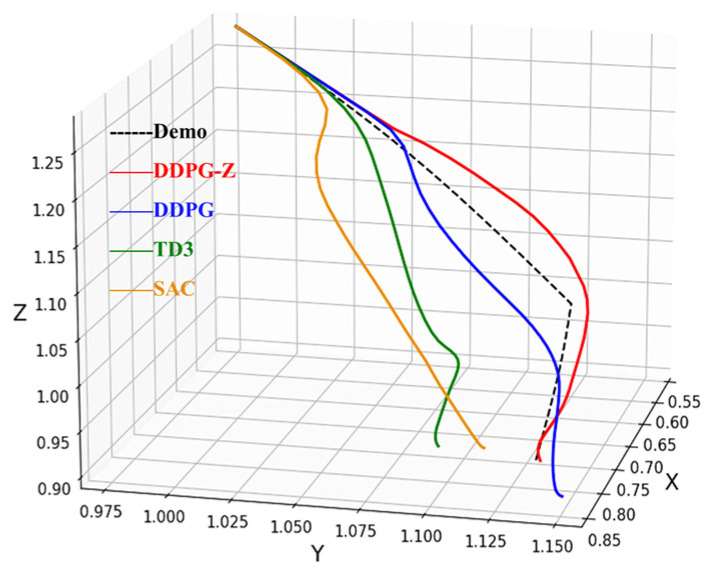
Comparison of paths planned by each model and demonstration path in comparison tests.

In the simulation of 20 random episodes, the DDPG-Z model still achieved the best evaluation results, completing the fruit bunch collection task with a 100% success rate, and all metrics were optimal (Table 4). For the other three models, due to failing to reach a stable state within 500 training episodes, they performed poorly on all metrics, with DDPG only achieving a 15% success rate, and the remaining two models both at 0%. We randomly selected a placement location different from Figure 7 and compared the demonstration path with the paths planned by the four models. The results showed that the path planned by our model remained the smoothest and most similar to the demonstration path (Figure 10). In contrast, the paths of the other three models were less smooth and had a lower similarity to the demonstration path. However, DDPG was generally slightly better than SAC and TD3. Figure 8b showcases the different effects of fruit bunch placement by the robotic arm in the simulation environment according to the aforementioned paths, with our model exhibiting the closest performance to the demonstration effect near the contact point and the destination. At the destination, the DDPG model appeared to have a good placement effect due to a visual error; however, in reality, there was a high risk of collision between the end-effector and the fruit basket due to significant offsets on the X and Z axes. For the TD3 model, the end-effector was too high at the destination, risking damage to the fruit bunch upon dropping. The placement effect of the SAC model was the worst, severely encroaching on other placement positions.

In summary, our method significantly enhanced the model’s convergence performance and stability compared to the standard DDPG, learning a superior policy within just 500 episodes and demonstrating the capability to dexterously place fruit bunches with precision.

### 3.3. Application of Tomato Bunch Collection Paths

**Figure 11:**
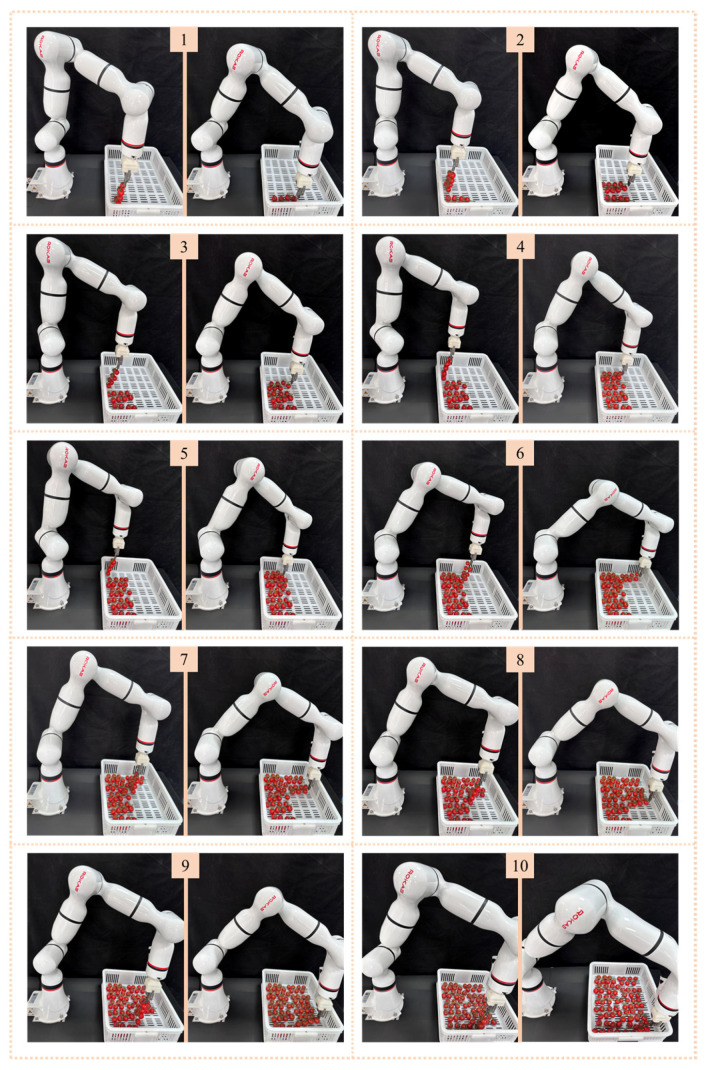
Performance of the ten paths planned by DDPG-Z in the real collection tasks. The numbers at the top of each group of pictures represent the different positions in the fruit basket.

In addition to the tests in the simulation environment, we also verified the ten paths planned by DDPG-Z using real fruit bunches and the tomato bunch collection mechanism described in Section 2.1. Observations indicated that the placement effects of the real robotic arm were largely consistent with the predicted results from the simulation environment, demonstrating dexterousness comparable to manual operation. Throughout the process, the robotic arm did not collide with the fruit basket, and the tomato bunches were placed gently in the fruit basket without experiencing free fall. Overall, the ten tomato bunches were placed in an orderly and neat manner (Figure 11).

Our study shares similarities with the work presented in [28], where the DDPG algorithm is integrated with expert experience to plan fruit-picking paths for the robotic arm. The findings from that study also validate the effectiveness of incorporating demonstrations in significantly enhancing the learning efficiency of the model. However, the demonstrations used in that study were generated by the RRT algorithm, and the reward function was simplistically defined as the distance between the current position of the robotic arm’s end-effector and the target position. Although this method is suitable for tasks without specific path shape requirements, it may not be sufficiently effective for complex tasks that necessitate the emulation of human-like flexible operations.

## 4. Conclusions

This study proposed a compliant path planning method to optimize the fruit bunch collection process of the harvesting robotic arm. The method realized the integration of human skills with DDPG and improved the flexibility of the robotic arm’s movement, thus avoiding the risk of damage to the fruit bunch during collection. In addition, the expert reward regression loss, dynamic step-length return, and prioritized experience replay strategies contributed significantly to enhancing the model performance. Under the same environment and parameters, our model’s convergence speed was at least 40% faster than that of the conventional DDPG, with more stable performance and paths that were closer to the demonstration paths. The results of ablation tests indicated that the introduction of demonstration data was most significant in enhancing model performance. Specifically, compared to the No demo model, the DDPG-Z model converges 25% faster, with a 10.3% improvement in the average reward after convergence and a 51.3% improvement in the average positioning accuracy of the destination. In summary, the method will contribute to instructing the harvesting robotic arm to learn complex human-like agronomic operations.

Nonetheless, the current planning process still has some limitations. Specifically, the whole planning process is open-loop, i.e., the model only performs path planning based on the pre-set placement positions without considering the dynamic changes in the actual operation. In practice, the differences in the movement accuracy of the robotic arm and the size of the tomato bunches may lead to mutual extrusion between the bunches. To address these limitations, future research will focus on introducing real-time feedback mechanisms, such as adding visual information to help the robot arm fine-tune its placement position, to further enhance its adaptability and flexibility in complex environments.

## Figures and Tables

**Table 1:** D–H parameters of the robotic arm.

Joint	Link Length (m)	Link Rotation Angles (°)	Link Offset (m)	Initial Angles of Joint (°)	Joint Rotation Range (°)
1	0	0	0.341	4.95	−170 to 170
2	0	−90	0	−6.87	−120 to 120
3	0.394	90	0	−7.67	−170 to 170
4	0	−90	0	79.63	−120 to 120
5	0	90	0.366	−0.96	−170 to 170
6	0	−90	0	107.18	−120 to 120
7	0.404	90	0	−2.94	−360 to 360

**Table 2:** The average reward of each model at the beginning and end of training.

Model	Avg. Reward	
	First 50 Episodes	Last 50 Episodes
DDPG-Z	−24.45	−8.18
No Demo	−44.98	−9.12
No PER	−33.84	−8.54
No DSR	−33.74	−8.92
No ERR	−37.12	−8.62

**Table 3:** The simulation results of the five distinct models in ablation tests.

Model	$avg.d_{1}\pm \sigma_{1}$ (m)	$avg.d_{2}\pm \sigma_{2}$ (m)	Success Rate	$avg.n_{step}\pm \sigma_{n}$
DDPG-Z	0.027 ± 0.002	0.019 ± 0.003	100%	25 ± 0
No Demo	0.041 ± 0.006	0.039 ± 0.011	50%	32.6 ± 6.7
No PER	0.038 ± 0.012	0.035 ± 0.003	70%	29.4 ± 3.4
No DSR	0.038 ± 0.015	0.037 ± 0.011	60%	36.3 ± 3.2
No ERR	0.041 ± 0.005	0.030 ± 0.005	75%	26.2 ± 0.5

**Table 4:** The simulation results of the four distinct models in comparison tests.

Model	$avg.d_{1}\pm \sigma_{1}$ (m)	$avg.d_{2}\pm \sigma_{2}$ (m)	Success Rate	$avg.n_{step}\pm \sigma_{n}$
DDPG-Z	0.027 ± 0.002	0.019 ± 0.003	100%	25 ± 0
DDPG	0.069 ± 0.026	0.049 ± 0.022	15%	37.1 ± 5.2
TD3	0.056 ± 0.031	0.078 ± 0.029	0%	40 ± 0
SAC	0.091 ± 0.030	0.057 ± 0.022	0%	38.1 ± 4.6

## Data Availability

The data that support the findings of this study are available upon request from the corresponding author. The data are not publicly available due to privacy or ethical restrictions.
